# Delayed outbreak detection: a wake-up call to evaluate a surveillance system

**DOI:** 10.11604/pamj.supp.2022.41.1.31161

**Published:** 2022-02-09

**Authors:** Lilian Bulage, Daniel Kadobera, Benon Kwesiga, Steven Ndugugwa Kabwama, Alex Riolexus Ario, Julie Rebecca Harris

**Affiliations:** 1 Uganda National Institute of Public Health, P.O Box 7272, Kampala, Uganda,; 2 African Field Epidemiology Network, Kampala, Uganda,; 3 Ministry of Health, Kampala, Uganda,; 4 College of Health Sciences, Makerere University School of Public Health, Kampala, Uganda,; 5 United States Centers for Disease Control and Prevention, Kampala, Uganda,; 6 Division of Global Health Protection, Center for Global Health, US Centers for Disease Control and Prevention, Atlanta, United States of America

**Keywords:** Case Study, malaria, surveillance system evaluation, outbreak detection, Uganda

## Abstract

During May, 83 of the 120 districts in Uganda had reported malaria cases above the upper limit of the normal channel. Across all districts, cases had exceeded malaria normal channel upper limits for an average of six months. Yet no alarms had been raised! Starting in 2000, Uganda adopted the World Health Organization (WHO) Integrated Disease Surveillance and Response (IDSR) strategy for disease reporting, including for malaria. Even early on, however, it was unclear how effectively IDSR and DHIS2 were being used in Uganda. Outbreaks were consistently detected late, but the underlying cause of the late detection was unclear. Suspecting there might be gaps in the surveillance system that were not immediately obvious, the Uganda FETP was asked to evaluate the malaria surveillance system in Uganda. This case study teaches trainees in Field Epidemiology and Laboratory Training Programs, public health students, public health workers who may participate in evaluation of public health surveillance systems, and others who are interested in this topic on reasons, steps, and attributes and uses the surveillance evaluation approach to identify gaps and facilitates discussion of practical solutions for improving a public health surveillance system.

## How to use this case study

**General instructions:** case studies in applied epidemiology allow students to practice applying epidemiologic skills in the classroom to address real-world public health problems. The case studies are used as a vital component of an applied epidemiology curriculum, rather than as stand-alone tools. They are ideally suited to reinforcing principles and skills already covered in a lecture or in background reading. This case study has a facilitator guide and a participant guide. Each facilitator should review the Facilitator Guide, gain familiarity with the outbreak and investigation on which the case study is based, review the epidemiologic principles being taught, and think of examples in the facilitator´s own experience to further illustrate the points. Ideally, participants receive the case study one part at a time during the case study session. However, if the case study is distributed in whole, participants should be asked not to look ahead.

During the case study session, one or two instructors facilitate the case study for 8 to 20 students in a classroom or conference room. The facilitator should hand out part I and direct a participant to read one paragraph out loud, then progressing around the room and giving each participant a chance to read. Reading out loud and in turns has two advantages. First, all participants engage in the process and overcome any inhibitions by having her/his voice heard. Second, it keeps all the participants progressing through the case study at the same speed.

After a participant reads a question, the facilitator will direct participants to answer the question by performing calculations, constructing graphs, or engaging in a discussion of the answer. Sometimes, the facilitator can split the class to play different roles or take different sides in answering the question. As a result, participants learn from each other, not just from the facilitator. After the questions have been answered, the facilitator hands out the next part. At the end of the case study, the facilitator should direct a participant to once again read the objectives on page 1 to review and ensure that the objectives have been met.

**Prerequisites:** for this case study, participants should have received lectures or conducted readings in public health surveillance and public health system evaluations.

**Target audience:** trainees in the Uganda Field Epidemiology Training Program/ Public Health Fellowship Program, other Field Epidemiology and Laboratory Training Programs (FELTPs), public health students, public health workers who may participate in evaluation of public health surveillance systems, and others who are interested in this topic.

**Level of case study:** advanced

**Time required:** approximately 12 hours

**Language:** English

## Case study material

- Download the case study student guide (PDF - 444 KB)
- Request the case study facilitator guide

## References

[1] Ministry of Health Knowledge Management Portal (2021). The Uganda Malaria Reduction Strategic Plan 2014-2020.

[2] Talisuna AO, Noor AM, Okui AP, Snow RW (2015). The past, present and future use of epidemiological intelligence to plan malaria vector control and parasite prevention in Uganda. Malar J.

[3] Lukwago L, Nanyunja M, Ndayimirije N (2013). The implementation of Integrated Disease Surveillance and Response in Uganda: a review of progress and challenges between 2001 and 2007.

[4] District Health Information System 2 (DHIS2) (2021). Open Health News [Internet].

[5] CDC Updated Guidelines for Evaluating Public Health Surveillance Systems [Internet].

[6] ECDC (2014). Data quality monitoring and surveillance system evaluation - A handbook of methods and applications [Internet]. Eur Cent Dis Prev Control.

[7] Uganda Ministry of Health Home | Mtrac [Internet].

[8] UNICEF mTrac [Internet].

